# Slippery Alkoxysilane Coatings for Antifouling Applications

**DOI:** 10.1021/acsami.3c00555

**Published:** 2023-03-23

**Authors:** Henry Apsey, Donald Hill, Andrew R. Barron, Shirin Alexander

**Affiliations:** † Energy Safety Research Institute (ESRI), School of Engineering and Applied Sciences, Swansea University Bay Campus, Fabian Way, Swansea SA1 8EN, U.K.; ‡ Arizona Institute for Resilient Environments and Societies (AIRES), University of Arizona, Tucson, Arizona 85721, United States; § Department of Chemistry and Department of Materials Science and Nanoengineering, Rice University, Houston, Texas 77005, United States; ∥ Faculty of Engineering, Universiti Teknologi Brunei, Darussalam BE1410, Brunei

**Keywords:** antifouling, slippery surfaces, siloxane hydrophobic coatings, water contact angle, durable, transparent

## Abstract

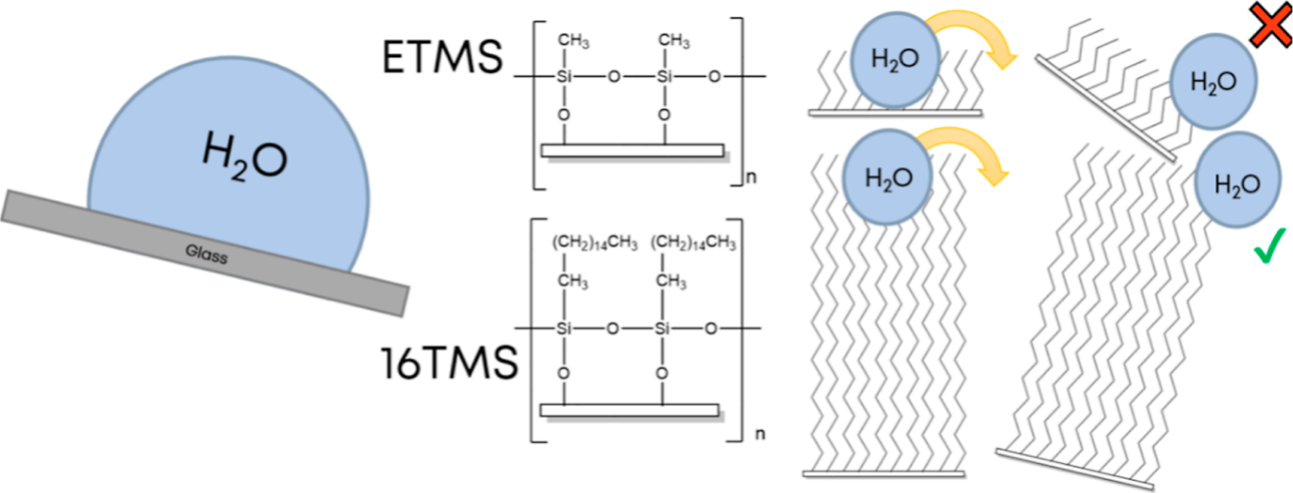

Herein, we report the wettability and antifouling behavior of a range of different siloxane coatings on plastic and glass substrates. The films investigated are prepared using trimethoxysilane precursors with different alkyl chain lengths (1–18 C atoms) in order to study how the nature of the hydrophobic group affects the different parameters used to characterize wettability (contact angles, sliding angles, and contact angle hysteresis). Atomic force microscopy analysis shows that the coatings possess low surface topography [root mean squared roughness (rms) < 50 nm] and are highly transparent as studied using UV–vis spectroscopy. The sliding properties of H_2_O, CH_2_I_2_, methanol, and ethylene glycol were observed to be strongly influenced by the chain length of the alkoxysilane precursor used. The coatings formed from the longer chain analogues show comparable water sliding angles to superhydrophobic surfaces. These coatings show similar performance to analogous alkoxysilane coating-bearing fluorinated groups, indicating that they could act as viable environmentally friendly alternatives to some of the fluorinated films that have been widely adopted. Furthermore, these surfaces are highly durable toward common forms of abrasion and are observed to show low adhesion toward synthetic feces, indicating that their utility extends further than repelling liquids alone. Consequently, these coatings could show promise for potential use in applications in the medical sector where fouling by biological mixtures leads to an unsustainable use of materials.

## 1 Introduction

While the discovery of superhydrophobic surfaces dates back to the 1930s,^1^ synthetic superhydrophobic surfaces have undergone significant research in the last 20 years, with the aim of producing surfaces with the properties of both (a) static water contact angles >150° and (b) sliding angles of below 10°. The generation of these superhydrophobic surfaces has relied significantly upon biomimicry to produce liquid repellent coatings and many synthetic analogues have relied heavily on fluorocarbon-based alternatives due to their low surface energy.^2,3^ Within nature, there are a wide range of surfaces which show interesting properties with extremes of wettability, from which we can learn and then design smart functional materials.^4^ These surfaces have potential application for a wide range of uses, including self-cleaning,^5^ anti-icing,^6^ anti-fouling,^7^ oil and water separation,^8^ and surface protection.^9^ However, superhydrophobic surfaces suffer from low mechanical and structural stability of their nanoscale structure, which leads to a loss of effectiveness.^10^

In addition to these low wettability surfaces, another class of coating has also been observed to show comparably low sliding angles toward water and other liquids. Slippery liquid-infused porous surfaces (SLIPs) provide a smoother surface by employing a microstructured pore surface which functions as a reservoir for lubricating fluid.^11^ Sliding of liquids on surfaces of this nature is facilitated by the lubricant which transports droplets away from the surface when they are slightly angled or tilted. These coatings possess surface roughness at much shorter length scales relative to most superhydrophobic coatings.^12,13^ This allows for greater transparency, which means they can be applied for a wider range of applications. The lubricant is held in place by the micro–nanoporous matrix.

SLIPs made from alkoxysilanes show a significant promise^14,15^ and all of this is possible without a fluoro group.^16^ The hydroxyl group chemically adsorbs to a hydroxyl surface, such as ceramics or metal oxides, through a sol–gel reaction and physically adsorbs onto surfaces such as plastics. However, this can be difficult to attach to a plastic surface as the hydroxyl groups need to be covalently bonded. Adjusting the alkyl chain length of the silane can increase the surface hydrophobicity as a result of stronger shielding of the surface by the chains.^17^ Cross-linkers can then form as a result of the loss of three methoxy leaving groups, which leads to a strong durable surface with increased chemical resistance to both hydroxyl groups and fatty acids or oils.^18^ Alkoxysilanes chemically adsorb onto hydroxylated surfaces (ceramics, silica etc). However, during the preparation of liquid-entrenched surfaces, excess alkoxysilane reacts through polycondensation reactions to generate a coating made up of polymers and oligomers, which show a low surface topography.

Advances that are more recent have come in the form of smooth surfaces, which are nonlubricated, unlike SLIPS. These surfaces show that liquids are capable of sliding on surfaces with low tilt angles (<20^°^) without oil and without surface topographies required to affect superhydrophobic/superomniphobic behavior.^16,19^ These surfaces display a reduced adhesion to solids compared to smooth equivalents without a lubricating layer, leading to improved anti-fouling capabilities.^20^ A key example of this was reported by Wang et al. who produced so-called liquid-entrenched smooth surfaces (LESSs) using dimethyldimethoxysilane to reduce the adhesion of viscoelastic solids such as artificial feces by up to 90%. The highly robust antifouling LESS was studied for application in toilets and was observed to potentially reduce water consumption by 10% in comparison with untreated surfaces and also showed lower bacterial fouling and odor build-up.^19^ Dong et al. developed a similar methodology using a methyltrimethoysilane-based solution, which created a film that showed great repellence toward a range of liquids (water, hexadecane, diiodomethane, and paints), with sliding angles as low as 4^°^. The anti-fouling properties were shown to apply across a range of surfaces and showed high durability and thermal stability.^16^

**Scheme 1:**
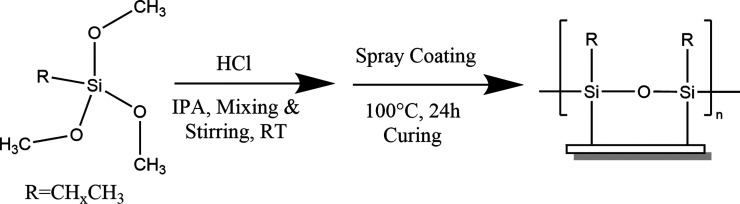
Overview of Reaction Procedure, x Varies from 1 to 18

The study we are reporting, here builds upon these recent reports with an increased focus in the antifouling properties to liquids and feces in oiled and dry conditions. Our investigation focuses on a range of coatings formed from alkoxysilane precursors, bearing different alkyl chain lengths (1–18 C atoms), in order to examine the effect of chain length on LESSs. In addition to looking at the effects of oiling, these coatings are compared to the fluorinated and hydrocarbon analogues to further understand and develop the scope of this emerging methodology.^21^ A summary of this approach is shown in Scheme 1.

## 2 Materials and Methods

### 2.1 Materials

Alkyl and fluoroalkyl trimethoxysilanes (TMSs); (methyltrimethoxysilane (MTMS), ethyltrimethoxysilane (ETMS), butyltrimethoxysilane (4TMS), decyltrimethoxysilane (10TMS), dodecyltrimethoxysilane (12TMS), hexadecyltrimethoxysilane (16TMS), octadecyltrimethoxysilane (18TMS), decyltriethoxysilane (10EMS), 1*H*,1*H*,2*H*,2*H*-Perfluorodecyltriethoxysilane (PFTES), and silicon oil were purchased from Fisher Scientific (Alfa Aesar). Concentrated hydrochloric acid (HCl) (34%), ethanol (99.5%), and isopropanol (99.5%) were purchased from Merck Life Sciences. Glass slides were acquired from Avantor (VWR), and plastic samples are composed of 5-ply ethylene vinyl acetate/ethylene vinyl acetate/polyvinylidene dichloride/ethylene vinyl acetate/ethylene vinyl acetate.

### 2.2 Coating Methods

Plastic samples (75 μm thickness) were cut into 50 × 50 mm^2^ pieces and washed with ethanol and isopropanol to remove surface contaminants. Glass slides of 75 × 26 mm^2^ were washed with acetone and ethanol to remove surface contaminants and expose hydroxyl groups. Each carbon chain length alkoxysilane (12 mmol) dissolved in 22.5 mL of isopropanol and 0.85 mL concentrated HCl was added drop-by-drop and stirred for 24 h. Various application methods (including cloth, sponge, spraying) were tested to find the optimum for our conditions. Spray-coating was found to be the most effective method of generating good-quality films. Coatings were sprayed onto plastic and glass slides using a compressed air propellant spray gun with a pressure of 20 psi. The coatings were then cured for 48 h at 65 °C to polymerize the alkoxysilane via a sol–gel reaction.^22^ This temperature was chosen to avoid the plastic melting and deforming and was used for annealing on glass substrates in order to be consistent. Selected surfaces were then dip-coated in silicone oil (180 cSt) to create an overcoat lubricant layer.

### 2.3 Characterization

#### 2.3.1 Surface Analysis

The topography and surface roughness of samples were determined by atomic force microscopy (AFM), using a JPK Nanowizard in the tapping mode. Surface roughness values for the films were measured from three different 20 × 20 μm^2^ areas of the surfaces. Roughness values were averaged from three locations and the uncertainties are the standard deviation of the mean. X-ray photoelectron spectroscopy (XPS) was performed using an Axis Supra XPS fitted with a monochromated Al K_a_ source and large area slot mode detector (ca. 300 × 800 μm^2^ analysis area). Spectra were recorded using a charge neutralizer to limit differential charging and binding energies were calibrated to the main hydrocarbon peak (BE 284.8 eV). The XPS data was analyzed using CASA software with Shirley backgrounds. Ultraviolet–visible (UV–vis) spectroscopy was run using a Perkin Elmer Lambda 365 in the transmission mode. Scanning electron microscopy (SEM) was performed using a Hitachi Field-Emission S-4800 scanning microscope with an accelerating voltage of 1.0 kV.

#### 2.3.2 Wettability

Contact angle and tilting angle measurements were determined from a Krüss DSA 25 with an IDS UI-306xCP-M camera using Advance software. A Young Laplace fitting method was used to calculate the contact angles. Distilled water (Millipore, 18 MΩ cm) and diiodomethane (Merck Life Sciences) were used as test liquids. Measurements were taken on 3 different locations across the sample using 5 μl droplets and a dosing rate of 2.66 μL s^–1^. The dropping height is the distance between the needle tip and the surface, which was maintained at 15 mm throughout this study. Advancing and receding contact angle measurements were performed through dosing a 4 μL water droplet with water at a rate of 0.5 μL s^–1^ until its volume reached 34 μL. Water was then removed from the droplet at a rate of 0.5 μL s^–1^ until its volume returned to 4 μL. Advancing and receding water contact angles were measured as the volume of the droplet changed during the process. The tilting rate used was 120°/min. The measurements were carried out under ambient conditions. The sliding angle was defined as the angle where the droplet leaves the frame of the camera. Water and diiodomethane were used as test liquids for static contact and tilting angle measurements. Methanol and ethylene glycol/water mixes were used to study how surface tension affected the wettability of the surfaces.

#### 2.3.3 Synthetic Feces Testing

Synthetic feces was produced by an adapted method from Wang^19^ and Wignarajah.^23^ Fecal matter contains proteins, fats, fiber, bacterial biomass, inorganic materials, and carbohydrates with vastly differing compositions and ratios between different people.^24^ The water content in feces can range between as much as 63–86% by mass.^25^ In our experiments, 4–20 g of mixtures were made up using 75% wt water, which is classified as Type 4 on the Bristol stool scale.^24^ The balance was made up using other materials, in order to simulate fecal composition. These were as follows: yeast (7.5% wt), Cellulose (2.5% wt), Psylum husk (4.4% wt), miso paste (4.4% wt), almond oil (5% wt), and inorganics (5% wt). In order to test the adhesion of the mixture onto the surfaces, one leveled spatula weighing approximately 1 g was dropped onto the substrates from a height of 25 mm. The tilting angles were then measured in the same manner as for the liquid droplets. In separate experiments, larger quantities (5 g) of the mixture were also applied to glass samples held at 45°. The mixture was dropped from a height of approximately 5 cm to test performance at a static angle of the substrates.

## 3 Results and Discussion

Coating of surfaces with alkoxysilanes to generate cross-linked polymeric films has been used to great effect in recent literature^26,27^ to create low wettability coating that displays low surface roughness. This methodology relies on catalytic amounts of water and the presence of an acid to allow hydrolysis and subsequent polycondensation of the alkoxysilane precursors via a sol–gel reaction. If the substrate is also hydroxylated (glass, ceramic etc), there is also further opportunity for the polymers and oligomers to chemically adsorb onto the surface by further condensation reactions (Scheme 1). Evaporation of the solvent alcohol leads to the generation of a polymeric film, a proportion of which may be chemisorbed depending on the nature of the substrate used.^28^

**Scheme 2:**
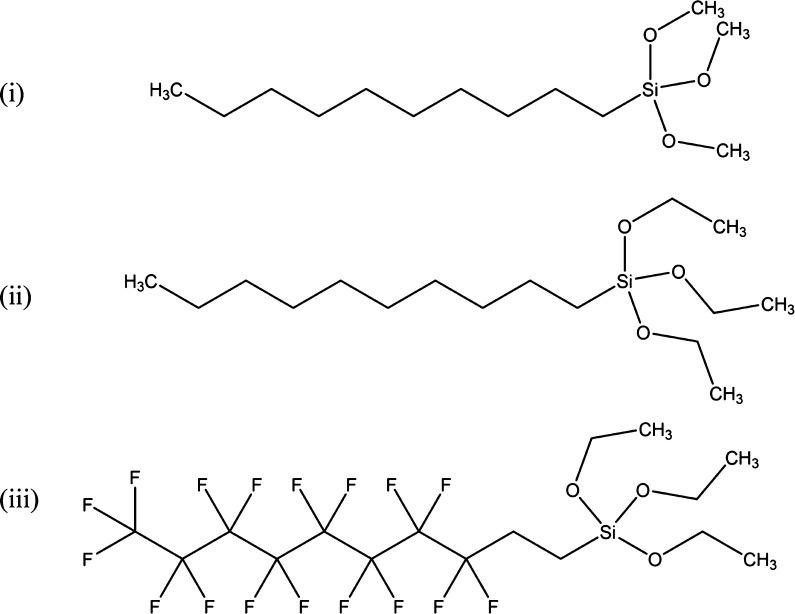
Siloxane Reagents Used to Examine the Effects of Fluorination and the Nature of the Alkoxy Group

In order to examine whether and/or how fluorination and alkoxy groups affect the wettability of the siloxane films, a selection of three different 10 C chain length alkoxysilane coating materials were chosen as model compounds for comparison, due to their similarity and availability (Scheme 2). Alkoxysilane coatings formed from decyltrimethoxysilane (10TMS) (i), decyltriethoxysilane (10TES) (ii), and 1*H*,1*H*,2*H*,2*H*-perfluorodecyltriethoxysilane (PFTES) (iii) were applied to surfaces (glass and plastic) using the same experimental conditions. XPS (Figure S2) showed the expected photoelectron and auger peaks for the three films (Si, O, C, and F for PFTES), confirming the presence of the silanes on the surfaces following spray-coating. AFM showed that these films possessed similar surface roughness; however, the PFTES film showed a distinct morphology (Figure S1).

**Figure 1:**
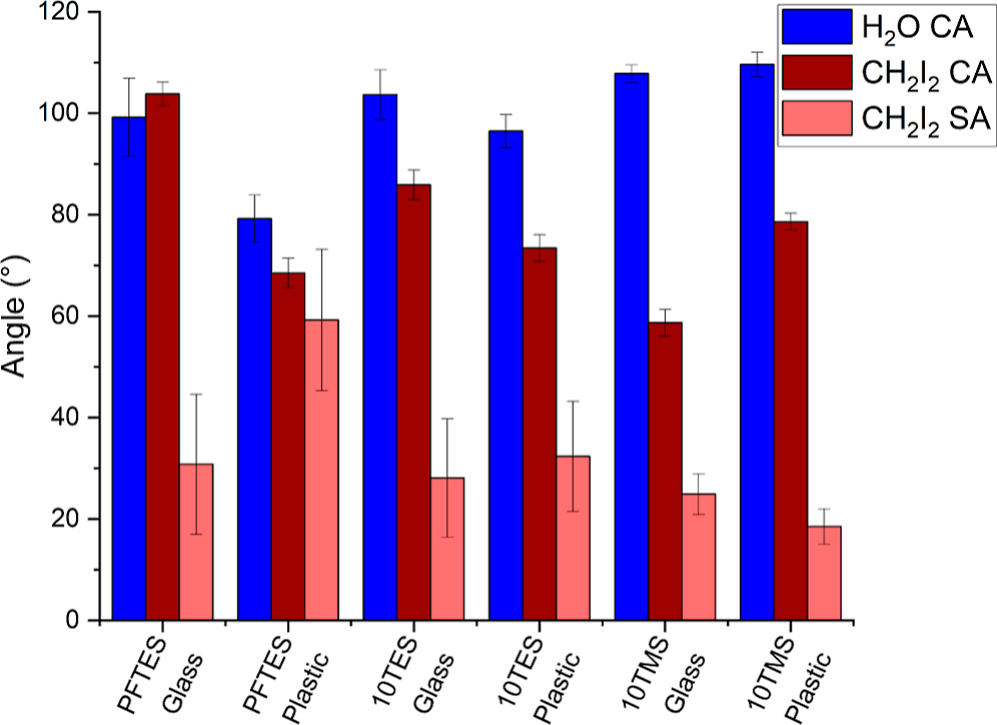
Graph comparing water contact angles as well as oil contact angle and sliding angles of 10 carbon chain fluorinated and hydrocarbon ethoxy and hydrocarbon methoxy silane films.

Interestingly, it is observed in Figure 1 that these films showed similar water contact angles (CAs) (CAs ∼100^°^, the dynamic CAs and contact angle hysteresis (CAH) are given in Supporting Information, Table S1) and CH_2_I_2_ CAs (∼80–100^°^). It should be noted that low water and oil CAs are due to the smooth and low surface topographies of these surfaces once annealed (Figure S1). No water sliding angles (SA) were observed for these samples due to the high CAH, but the diiodomethane SA were around 30^°^, despite the PFTES film containing fluorinated groups (surface free energy decreases in the sequence CF_3_ < CF_2_H < CF_2_ < CH_3_ < CH_2_).^29−31^ This suggests that the nature of the alkoxy substituents here does not have a large bearing on the film quality and also that less environmentally harmful alkoxysilanes can be used as precursors without significantly compromising the wettability. As a consequence of this, methoxysilane-bearing alkyl groups containing 1–18 C atoms (1, 2, 4, 10, 12, 16, and 18 TMS) were chosen to study how varying the carbon chain length affects the wettability of the films (Sections 3.1 and 3.2).

### 3.1 Surface Morphology and Composition of 1, 2, 4, 12, 16 and 18 TMS Coatings

**Figure 2:**
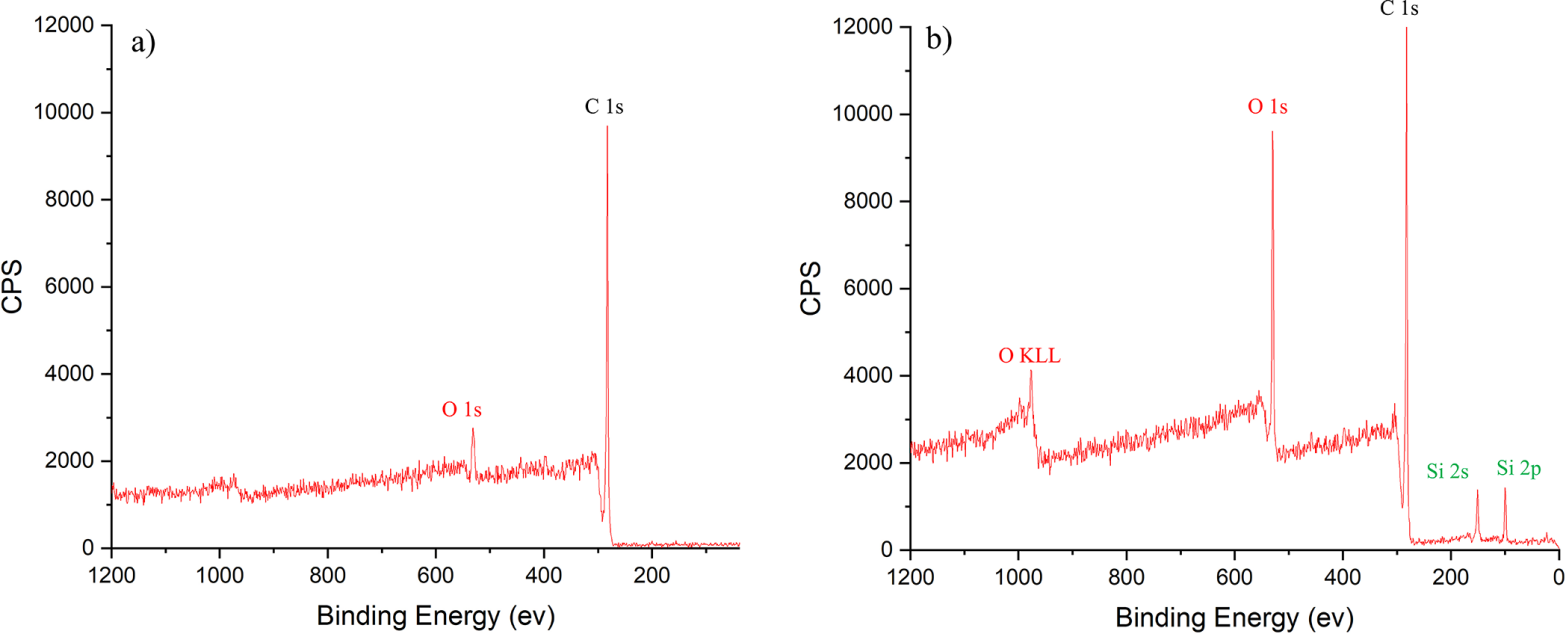
XPS spectra of (a) uncoated plastic film and (b) 16TMS-coated plastic.

The coatings of various chain length TMS were characterized on plastic samples to help understand the chemical composition and surface morphology of the coatings. The surface chemical composition of these surfaces was studied using XPS. The surface of the plastic samples were observed to be made up of largely carbon as is shown in uncoated plastic and 16TMS in Figure 2a and PFTES in Figure S2, with smaller photoelectron peaks ascribed to oxygen and nitrogen. By comparison, siloxane films of 16 TMS show photoelectron peaks ascribed to silicon and larger amounts of oxygen on the surface, providing evidence of film formation (Figure 2b) The atomic percentage of the elements calculated using the XPS data is shown Table S2).

**Figure 3:**
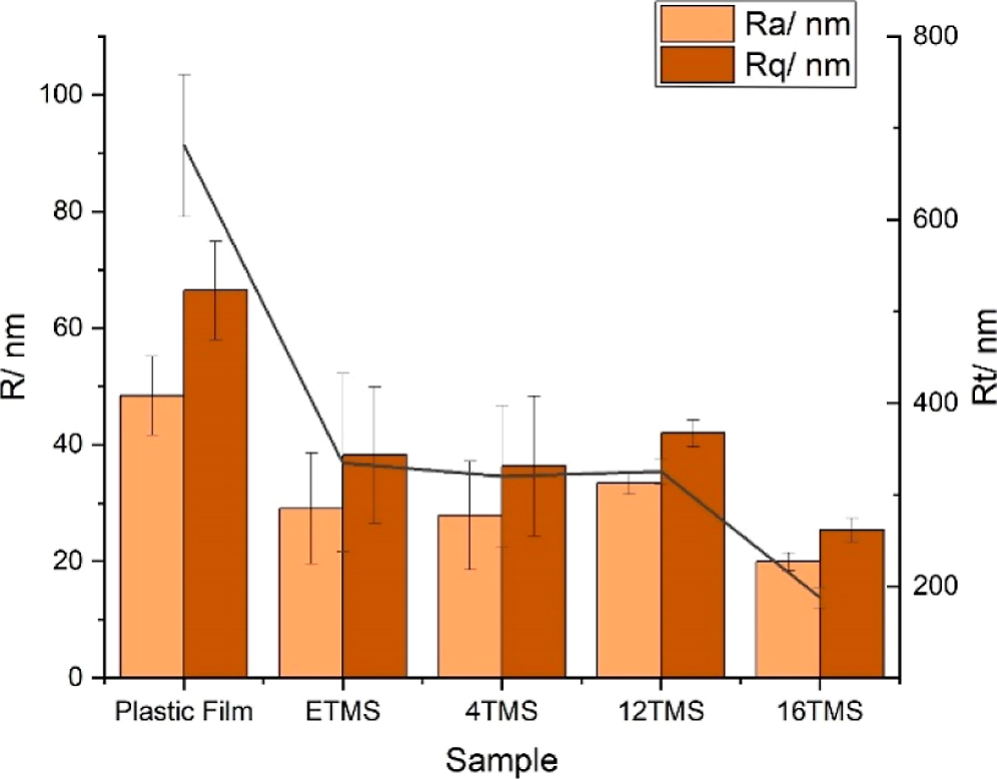
Plot comparing the roughness parameters: roughness average (*R*_a_), root mean squared (rms) roughness (*R*_q_), and maximum height of profile (*R*_t_) of selected surfaces.

The roughness of the films was measured using AFM, where it was observed that the coatings possessed a slightly lower topography than the as-received plastic (Figure 3 and Table S3). TMS coating-bearing alkyl groups ranging from 2 to 12 C atoms were observed to show similar surface roughness parameters, whereas the 16 C coating showed a slightly lower topography still. This could suggest that longer chain alkoxysilanes create smoother films. AFM images of these surfaces are displayed in Figure S3a–f. Additionally, AFM (see Table S3, and Figure S3g,h) and SEM (Figure S4a–c) were performed on film ETMS and 16 TMS before annealing to show the films at a further stage of the manufacturing where we observed increased roughness relative to the uncoated plastic. However, in both cases films appear smoother after annealing. The 16 TMS film (Figures S3h and S4c) undergoes substantial reorganization during annealing which leads to a far smoother film (Figures S3e and S4d). By comparison, the ETMS (Figures S3g and S4a) shows a far smaller reduction in surface roughness after annealing (Figures S3b and S4b), which could suggest less reorganization takes place. Optically, the films were observed to show a similar transparency to the as-received plastic and glass (Figure S5). This was confirmed by UV–vis spectroscopy, where the siloxane films showed a similar transmission of light within the visible range (Figure S6a,b). The transparency of the coatings is in line with the low surface roughness of the films observed during the AFM analyses.^32^

### 3.2 Coating Wettability

**Figure 4:**
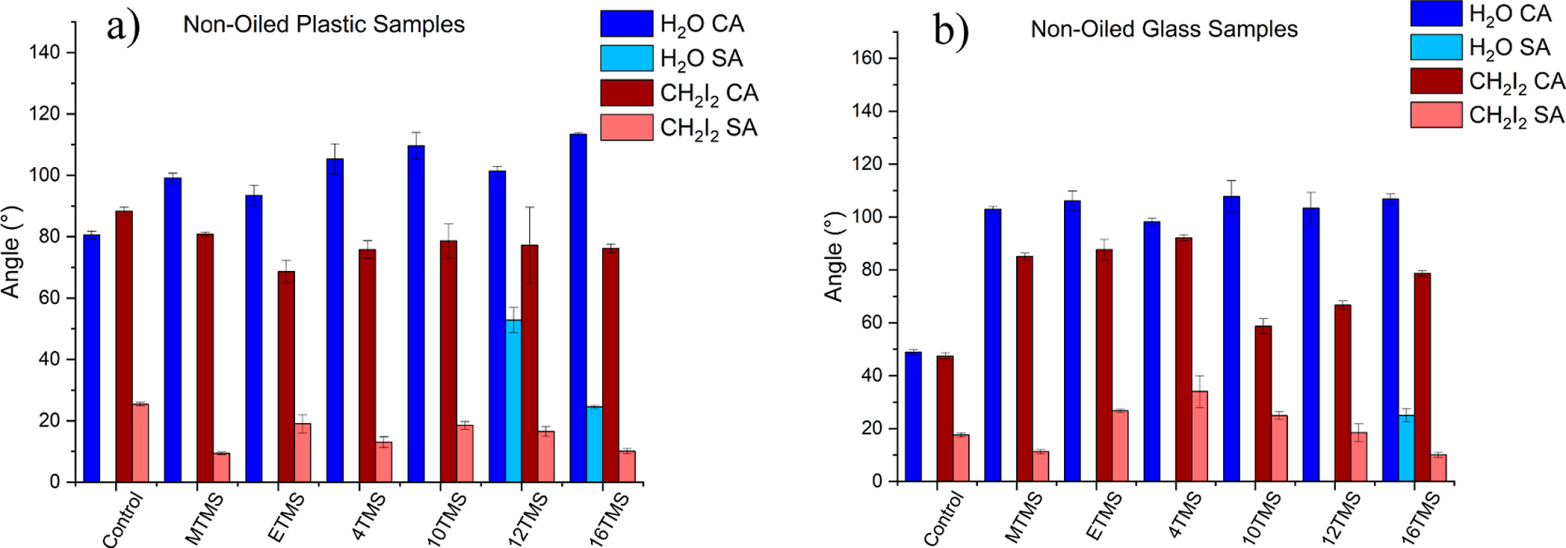
Graph showing static water and diiodomethane contact and sliding angles of (a) plastic and (b) glass coated with various chain lengths of silane.

The wettability of the surfaces was preliminarily studied using water and diiodomethane to investigate the interactions between the films and polar and non-polar liquids. Uncoated plastic samples were observed to show a water contact angle of about 80^°^ and a diiodomethane contact angle of 88^°^. By comparison, the water contact angles of siloxane films on plastic-bearing alkyl groups between 1 and 16 atoms were found to vary between approximately 93–113^°^, with no apparent relationship established between the chain length and the wettability (Figure 4a). The modest increase in hydrophobicity is perhaps not surprising because AFM showed that the films show smooth topographies, similar to the substrate. Diiodomethane contact angles of the films were observed to be similar and, in some cases, lower than the uncoated substrate (Figure 4a). Lower diiodomethane sliding angles were recorded on the siloxane films, relative to the as-received plastic, indicating that the coatings reduced the adhesion of the organic liquid onto the surface. By comparison, it was observed that water droplets did not slide on the as-received plastic, and on the siloxane films where the chain length was less than 12 C atoms (Figure 4a). This is contrary to what has previously been reported where low water sliding angles have been reported for siloxane coatings possessing methyl groups as the alkyl substituent.^16^ However, it is possible that differences between the formulation of the spraying suspension and the annealing temperature may account for the variation observed here.^16^ It was not possible to heat the plastic further because of its low melting temperature (<90 °C). Rolling of water droplets on the films was only realized when applying the 12 TMS coating onto the plastic. However, only a modest sliding of about 53^°^ was observed, indicating that the adhesion between the liquid and the coating was still relatively high. A substantially lower water sliding angle was observed for the 16 TMS coating (25^°^), which is ascribed to its lower surface energy and smoother topography relative to the other films. The 18 TMS coating was observed to be substantially more hydrophobic and showed a WCA of around 135^°^ and a water sliding angle of around 10^°^

Similar trends were observed when the coatings were deposited onto glass slides. Siloxane coatings bearing 1–12 C atoms showed water CAs ranging between 98 and 108^°^, substantially increased from the as-received glass (∼50^°^) (Figure 4b), again with no apparent trend between chain length and contact angle. Diiodomethane contact angles were also observed to be similar, although slightly lower than the contact angles of the films when they were applied to plastic (Figure 4a). In addition, similarly to the films on plastic, diiodomethane sliding angles were observed for all of the films. However, substantially higher tilt angles were observed for the 2, 4 and 10 TMS coatings when applied to glass, relative to when they were deposited on the plastic substrate (25–34° on glass versus 10–18° on plastic). As with the plastic specimens, water droplets were not observed to slide on the siloxane films on glass where the chain length ranged between 2 and 12 C atoms. Increasing the chain length further was observed to affect water droplet sliding and the 16 and 18 TMS coatings were observed to show sliding angles of approximately 25 and 5°, respectively (Figure 4b.). The lower adhesion of the longer chain analogues is ascribed to the lower film topography and increased hydrophobicity of the longer alkyl chains.^33^ Interestingly, the 18 TMS film was observed to show superhydrophobic Cassie–Baxter wetting on glass and show WCAs of >150^°^. However, unlike the others, this coating was observed to be white and only discrete areas of the film were able to cure and become transparent after annealing, which could suggest that the temperature of annealing was insufficient to cure the 18 TMS films uniformly. As the data were not reproducible for this coating; consequently, it was decided not to include 18 TMS in further experimentation or discussion in this study.

Advancing, receding, and hysteresis water contact angle measurements (Table 1) were also performed on the siloxane films on the glass in order to probe the surfaces’ heterogeneity.^34^ MTMS films were observed to show the lowest contact angle hysteresis (CAH), indicating that they showed the greatest surface homogeneity. By comparison, siloxane films bearing 12 and 16 C alkyl chains, and ETMS films were observed to show slightly higher CAH (ca. 16^°^) showing that the surfaces were more heterogenous. Films formed from the 4 TMS and 10 TMS coatings showed substantially higher CAH (22 and 40°, respectively). Taken together, these results suggest that there is a working window for the alkoxysilanes that can be used to create homogenous surfaces because greater surface homogeneity is observed when methylated and longer chain alkoxysilanes are used as film precursors. This behavior can be explained because films formed from molecules bearing short and medium length (<12 C atoms) show greater molecular disorder, which gives rise to larger CAH and hence greater adhesion to liquid droplets.^35,36^ Furthermore, the MTMS film, bearing only a methyl substituent could display the lowest CAH because it is smaller and therefore pack effectively alongside neighboring groups. By comparison, the longer alkyl groups have the capacity to exist in a larger number of possible conformations, which gives rise to a greater disorder and hence larger CAH.

**Table 1:** Dynamic Wettability Data and Water Sliding Angles of the Siloxane Films on Glass Substrate

	non-oiled			oiled		
film	advancing angle/°	receding angle/°	hysteresis/°	advancing angle/°	receding angle/°	hysteresis/°
MTMS	102 ± 1	92 ± 2	10	73 ± 3	66 ± 4	7
ETMS	99 ± 2	83 ± 2	16	75 ± 1	68 ± 2	7
4TMS	103 ± 4	63 ± 5	40	87 ± 3	79 ± 5	8
10TMS	99 ± 2	77 ± 2	22	96 ± 2	84 ± 1	10
12TMS	109 ± 2	92 ± 4	17	87 ± 4	65 ± 2	22
16TMS	108 ± 2	91 ± 2	16	98 ± 3	89 ± 4	9

**Figure 5:**
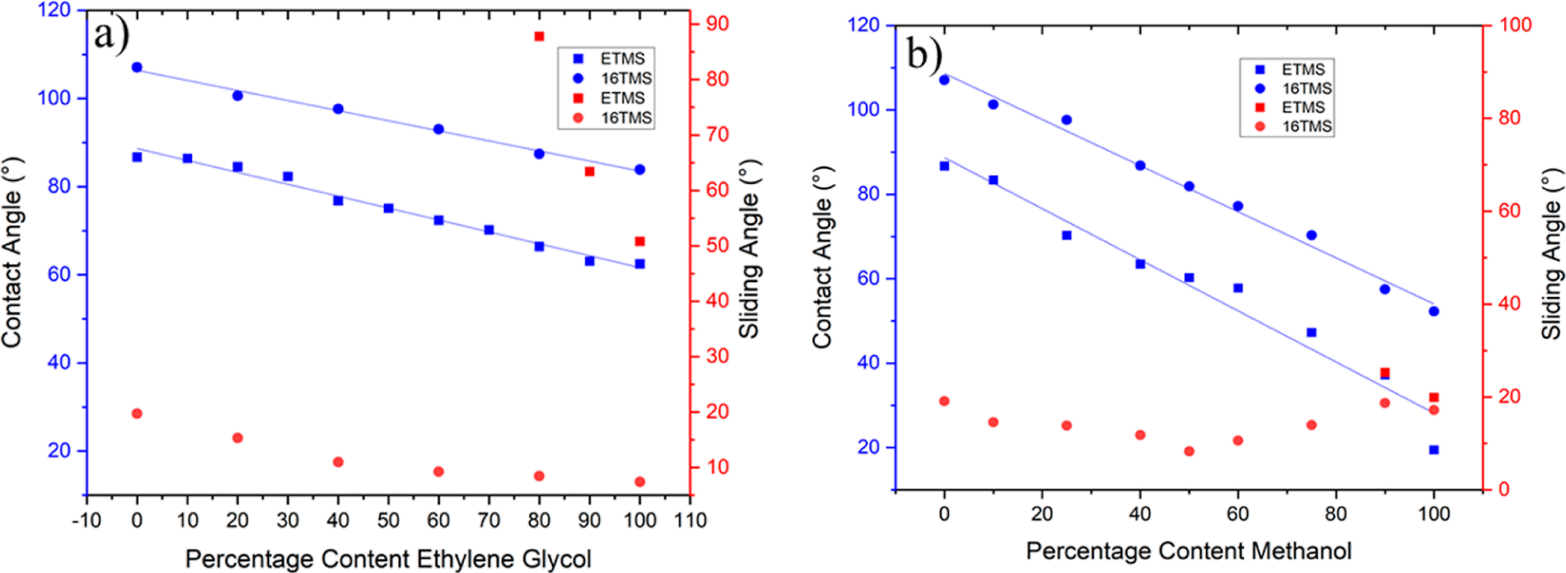
Relationship between the contact and sliding angles and percentage of various liquid droplets containing (a) water/ethylene glycol and (b) water/methanol mixtures for the ETMS and 16 TMS coatings.

The wettability of the siloxane films with lower surface tension liquids was investigated to further study the efficacy of the ETMS and 16 TMS coatings on glass samples. In these experiments, the volume fraction of ethylene glycol in ethylene glycol/water mixtures was varied in order to steadily lower the surface tension of the mixture from that of water, 72.8, to 48.2 m Nm^–1^, the surface tension of ethylene glycol.^37−39^ As anticipated, lowering the surface tension was observed to reduce the contact angles of both of the types of films (Figure 5a).^40,41^ Droplet sliding angles were observed for all of the water/ethylene glycol mixtures on the 16 TMS film. However, the sliding angles were observed to drop from approximately 25 to 5^°^ as the ethylene glycol content was increased from 0 to 100%. This behavior is ascribed to the higher density of ethylene glycol versus water (1.1 g/mL versus 1.0 g/mL), which facilitates droplet rolling on inclined surfaces.^42^ By comparison, droplets of pure ethylene glycol and mixtures containing at least 80% ethylene glycol only were observed to slide on the ETMS surface at substantially higher tilt angles (>50^°^), providing further evidence of the greater efficacy provided by 16 C chain. Droplets containing at least 70% ethylene glycol were observed to be more adherent and did not slide, even after tilting to 90^°^. Reductions in the contact angles were also observed for the films where the percentage content of methanol was increased in droplets containing mixtures of water and methanol. Similar to the water/ethylene glycol mixtures, this behavior is ascribed to the reduction in surface tension due to increasing the amount of the lower surface tension liquid. Lower contact angles were observed on both types of film than for the ethylene glycol/water mixtures because methanol has a lower surface tension than ethylene glycol (∼22.5 m Nm^–1^ versus 48.2 m Nm^–1^). Interestingly, it was observed that water/methanol droplets would slide on the 16 TMS film with no apparent trend between the methanol content and the sliding angle, whereas droplets containing only high methanol contents (>90%) could slide on the ETMS film. This data further displays the robustness of the 16 C chain for repelling liquid mixtures with a wide range of surface tensions.

### 3.3 Investigation of Coating Antifouling Properties toward Artificial Feces

Previous works carried out by Wang et al. have shown that siloxane films show excellent antifouling properties towards viscoelastic solids such as synthetic feces.^19^ Consequently, surfaces coated with siloxane films could have great utility as water-saving coatings for toilets and for other applications across the medical sector where surface fouling with mixtures of this nature poses problems. Human feces is very sticky and easily adheres to surfaces causing issues with contamination and increased water usage. As a result, it is highly desirable to investigate coatings that show repellence to highly adhesive solid/liquid mixtures such as this. To date reports studying the antifouling behavior have focused almost entirely on surfaces that are repellent to liquids, despite studies investigating properties such as density and viscosity being investigated therein.^43^ Wang and co-workers largely investigated the antifouling properties of synthetic feces on siloxane films that had been coated with a layer of oil to improve their lubricity.^19^ In this study, we examine what conditions around which the oil layer is necessary in relation to the chain length of the alkoxysilane used to form the films.

For this, an additional lubricant layer was applied onto the films using silicone oil. The sliding behavior of the mixture on these surfaces was then tested in the same manner as for the dry films. Previous studies have employed oil lubrication to improve the efficacy and lifetime of the underlying film, whereby the oil layer reduces contact between the solid material and surface features of the coating. Although protective, surface oiling increases the environmental impact of this methodology and increases the complexity associated with fabricating these surfaces. Consequently, we decided to investigate to what extent this layer contributed to the performance of our coatings.

**Figure 6:**
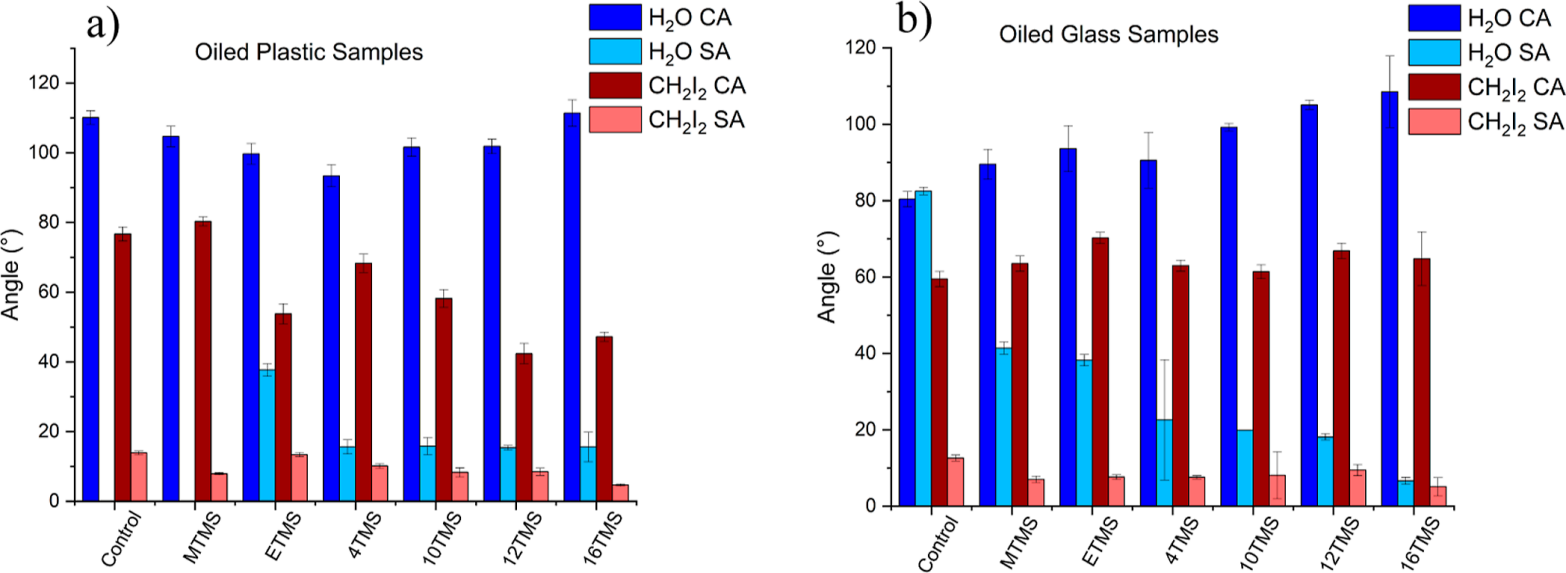
Graphs showing water and diiodomethane contact and sliding angles of (a) oiled plastic samples and (b) oiled glass samples.

Initially, we studied the wettability and sliding behavior of water and diiodomethane droplets on the oiled films in order to gain knowledge about how liquids behaved on the surfaces before any solid was added. It was observed that the silicone oil layer markedly reduced the diiodomethane sliding angle for the films on both glass and plastic substrates (Figure 6a,b, Table 1). Furthermore, the lubricant layer was also shown to reduce water sliding angles and cause water droplets to slide on the films formed from the shorter chain alkoxysilanes. This behavior is ascribed to the low shear properties of the oil and the immiscibility of the liquids and the lubricant layer.^12,44^ Interestingly, the chain length dependence on the wettability of the coatings was still observed after the application of the oil layer. This indicates that the nature of the alkoxysilane also has an effect here, despite the droplets having less contact with the coating.

**Figure 7:**
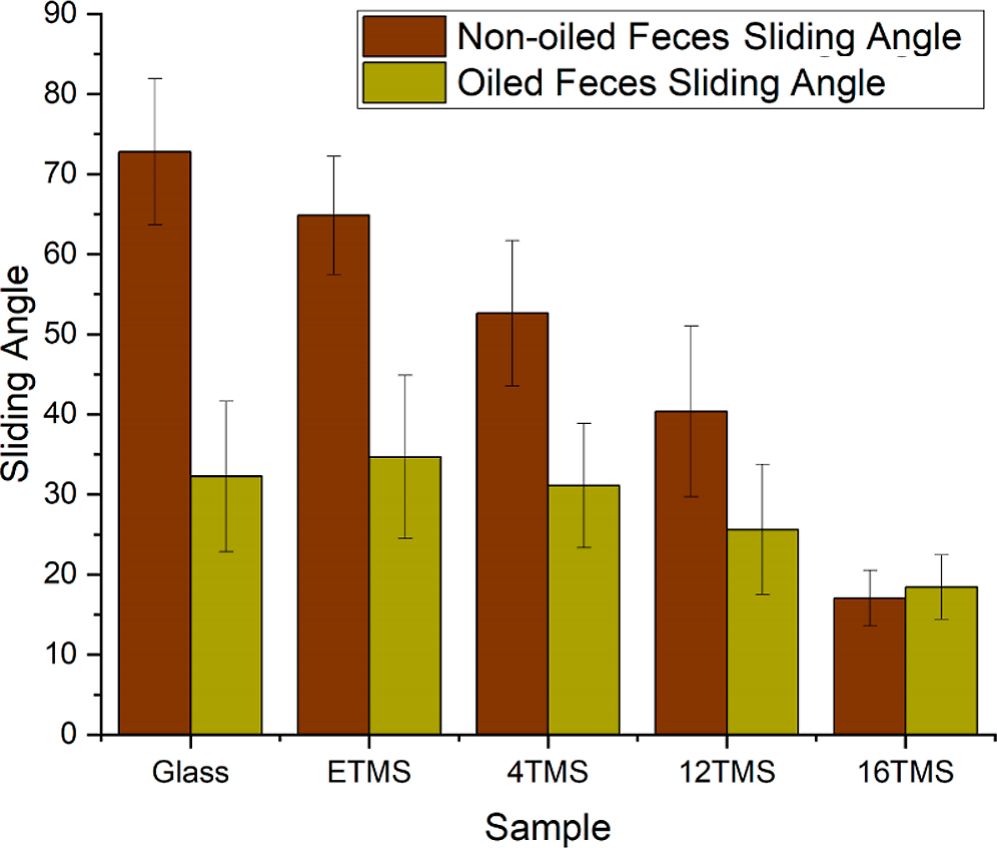
Graph showing feces SAs on glass and coated samples (oiled and non-oiled).

Following this, we investigated the sliding behavior of a synthetic feces mixture similar to that used by Wang et al. on our siloxane films on glass substrates. This was performed through measuring the angles at which the solid mixture was observed to slide off the surfaces.^19^ It was observed that large tilt angles were required to affect the sliding of the mixture on uncoated glass (∼73^°^) and large amounts of residue were observed on the surfaces following the experiments (Figure S7). Applying the siloxane coatings onto the slides were observed to create surfaces that showed lower sliding angles, which is ascribed to the low surface energy of the coatings and their smooth topography (Section 3.2). Interestingly, the sliding angles were observed to correlate with the alkyl chain length of the alkoxysilanes used to create the films, in line with the wettability data, and sliding angles as low as 17^°^ were observed when the mixture was applied to the 16 TMS coating (Figure 7). In addition, hardly any residue was observed on the coated surfaces indicating that the films perform well as antifouling coatings (Figure S7).

Applying the synthetic feces mixture to the microscope slides that had been coated with silicone oil was found to substantially lower the sliding angle, relative to the as-received glass (32^°^ versus 73^°^, Figure 7) and very little residue was observed on the glass after testing. This indicates that oil lubrication has a robust effect in its own right for repelling viscoelastic mixtures such as this. The sliding angles of the mixture on oiled ETMS and 4TMS films were observed to be similar to the oiled glass (31–35°), indicating that these coatings were redundant when the oil layer was also applied to the system. A reduction in the sliding angle was observed when the silicone oil was applied to the 12 TMS coating, where it fell to 26^°^ as is shown in Figure 7. Drawing from this result, and the wettability data of the oiled films, this reduction could suggest that the alkyl chain length was now sufficiently long enough to increase the mobility of the silicone oil and along with it the feces mixture. Increasing the chain length further was found to lead to a further reduction in sliding angle, where it dropped to 18° for the 16 TMS film. Interestingly, this value is very close to the angle recorded for the non-lubricated 16 TMS film (17^°^), suggesting that oiling siloxane films formed from alkoxysilanes with more than 16 C atoms does not further improve their performance. This result could suggest that there is sufficient mobility between the C chains of the 16 TMS film and the oils present in the feces mixture such that applying an additional layer of oil does not improve the performance. To test this hypothesis, the SAs of almond oil, which is present in the mixture, and peanut oil for comparison were recorded on the dry ETMS and 16 TMS films. In both cases, it was observed that the sliding behavior of these liquids was substantially better on films formed from the longer chain alkoxysilane (Table 2), which could suggest that the greater mobility of the feces mixture on the 16 TMS coating could be at least in part be due to its greater oleophobicity. We also performed a drop test which could be seen as the most representative test of real conditions to observe the amount of residue remaining on selected coatings (Figure S7).

**Table 2:** Table Comparing the Wettability Performance of Various Oils on ETMS and 16TMS Coatings

liquid	film	contact angle/°	sliding angle/°
silicon oil	ETMS	56 ± 4	21 ± 7
	16TMS	24 ± 2	6 ± 3
peanut oil	ETMS	72 ± 3	N/A
	16TMS	66 ± 4	39 ± 8
almond oil	ETMS	61 ± 5	59 ± 6
	16TMS	59 ± 6	23 ± 7

### 3.4 Coating Durability

**Figure 8:**
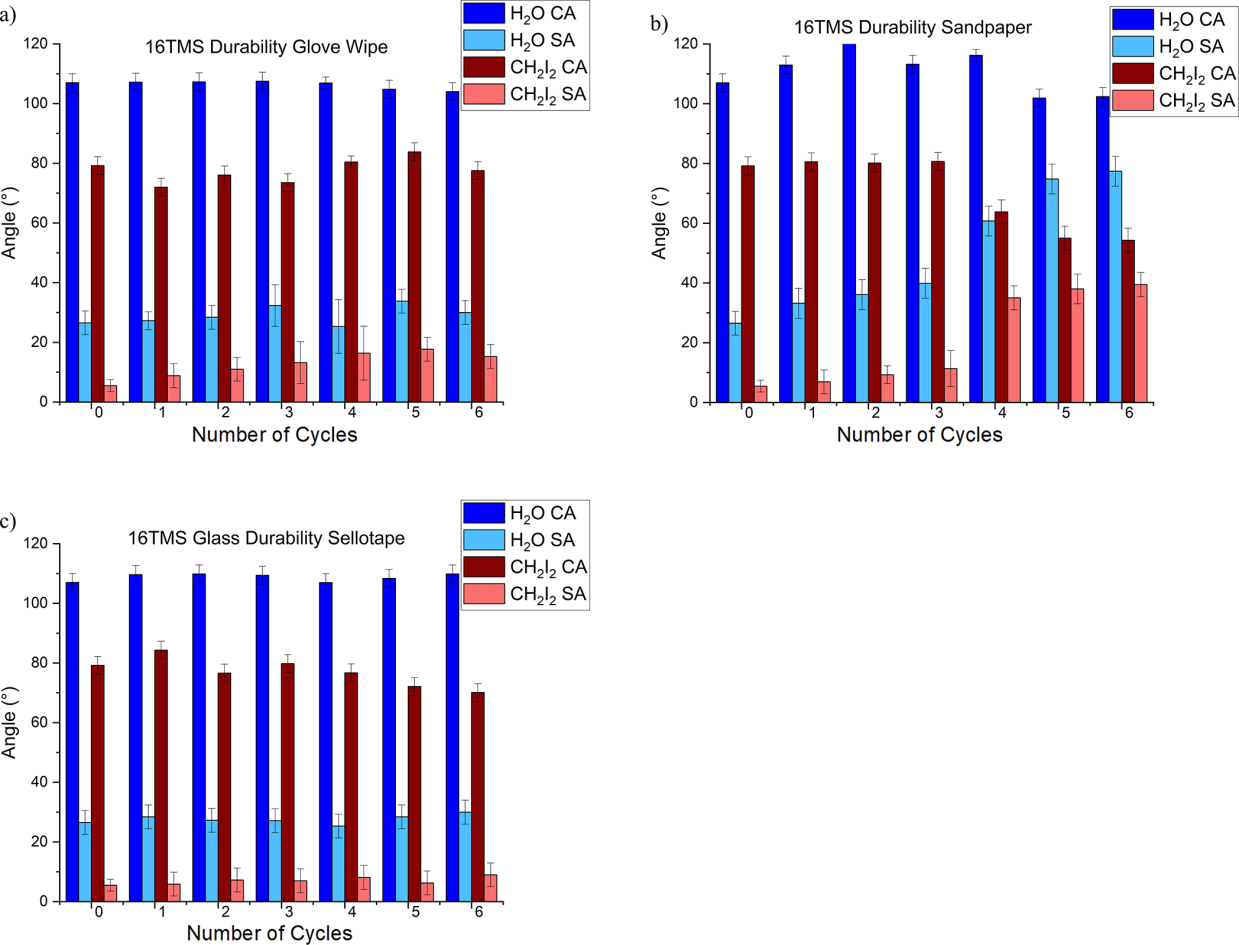
Graphs showing the wettability after scenarios of durability testing (a) repetitive wiping with a gloved finger, (b) rubbing of sandpaper, and (c) repetitive sticking and removal of Sellotape, upon 16 TMS-coated samples.

Investigations into the durability of the siloxane coatings were carried out on the ETMS and additionally 16 TMS coatings as model films, in order to assess their adhesion onto the substrates and also their potential utility in the conditions they are likely to experience if applied as antifouling coatings. Experiments were carried out where the films were subjected to glove wiping, tape-peeling, and more rigorous abrasion through rubbing with sandpaper. Repeated abrasion to ETMS and 16 TMS coated glass by nitrile gloves (0.11 mm thickness), silicon carbide sandpaper (450 grit), and tape peel tests using Sellotape (>3 N/cm Peel adhesion on steel, >15 N/cm tensile strength) was used with the pressure asserted by human fingers which has been observed that middle and index fingers typically exert forces ranging between 35 and 50 N.^45^ These abrasion methods were not observed to substantially reduce the water or diiodomethane CAs. The diiodomethane SAs on the ETMS coating were also not observed to vary significantly following these types of abrasion (Figure S8). Similarly, only modest increases in the diiodomethane SA were observed following the abrasion of the 16 TMS coating (Figure 8). In contrast, abrasion was found to have a marked effect on the water droplet SAs of the 16 TMS coating, whereby they were observed to increase sharply following glove wiping or rubbing using sandpaper and then become pinned to the surface following several cycles of abrasion. Tape peeling was observed to have a less damaging effect on the surface, whereby the water SAs were observed to increase less sharply, and droplets were still observed to slide off after six cycles of tape peeling. CAs and SAs of coatings over time (Table S4) and after deposition of multiple droplets were also recorded (Figure S9). Larger changes were observed for the shorter chain films, providing further evidence that the longer chain lengths are better candidates for use as antifouling coatings.

## 4 Conclusions

In this paper, we have reported a range of siloxane-coated surfaces that are fluorine free, slippery toward liquids and solids, and cheap to produce. This work builds upon the current published literature^16,19^ by providing a systematic study with a wide breadth of field, aiming to better understand the efficacy of these films by testing a range of oil free alkoxysilane coatings and artificial fecal matter testing. With a reduced environmental impact, these coatings show excellent liquid repellency toward water and organic liquids. This behavior can be further enhanced by the addition of a lubricant layer on top of the films to further improve their sliding angles. This was shown to provide the greatest improvement for the shorter chain films. It was observed that the longer chain films showed substantially greater performance relative to those formed from precursors containing shorter alkyl chains. This is likely to be due to a greater molecular disorder in the shorter chain films, as studied in related reports.^13^

Films formed from alkoxysilanes with longer chains were observed to show high performance on plastic and glass, suggesting that they could be of use in a broad range of applications, including coatings for medical devices, where surface fouling often poses major problems and reduces the lifetime of the product. Durability tests also showed that the films were resistant to most of the common forms of abrasion that they might encounter in everyday life (finger wiping, contact with adhesive tape etc), suggesting that they could perform well if applied in real-world situations. It was observed that the longer chains were able to facilitate sliding of a variety of test liquids (water, diiodomethane, and ethylene glycol) and also synthetic feces mixtures, to a larger extent, relative to films formed from shorter chain precursors, possibly as a result of lower surface topography and increased molecular disorder.^7^ The improved results of the feces testing show that with a silane coating it is possible to increase the lifetime of a product by reducing the fouling significantly, which has positive environmental implications, all while additionally removing the oiling layer for longer chain lengths, particularly above carbon number 16.

## Supporting Information Available

Photographs, wettability contact, sliding angles and graphs, AFM, UV–vis spectroscopy, SEM images of coated surfaces, and XPS results (PDF)
