# Supplementary material for: Slippery Alkoxysilane Coatings for Antifouling Applications
Source: ACS Appl Mater Interfaces. 2023 Mar 23;15(13):17353–63. doi: 10.1021/acsami.3c00555 (PMC10080537; doi:10.1021/acsami.3c00555)
Supplement: Supplementary file 1 — am3c00555_si_001.pdf [file am3c00555_si_001.pdf]

Supporting Information

Slippery Alkoxysilane Coatings for Antifouling Applications.

Henry Apsey[†], *Donald Hill*[†,] *Andrew R Barron*[†, ξ,ϕ,φ], *Shirin Alexander*[†*]

† Energy Safety Research Institute (ESRI), School of Engineering and Applied Sciences, Swansea University Bay Campus, Fabian Way, Swansea SA1 8EN, United Kingdom.

[ξ] Arizona Institute for Resilient Environments and Societies (AIRES), University of Arizona, Tucson, Arizona 85721, USA.

[ϕ] Department of Chemistry and Department of Materials Science and Nanoengineering, Rice University, Houston, Texas 77005, USA.

[φ] Faculty of Engineering, Universiti Teknologi Brunei, Brunei Darussalam.

* s.alexander@swansea.ac.uk

Table S1: Dynamic water contact angle data for the of 10 carbon chain fluorinated and hydrocarbon ethoxy and hydrocarbon methoxy silane films.

Sample	Advancing angle/ °	Receding angle/ °	Hysteresis/ °
**PFTES**	98.5	81.4	17.1
**10TES**	95.2	79.8	15.4
**10TMS**	98.5	76.9	21.6

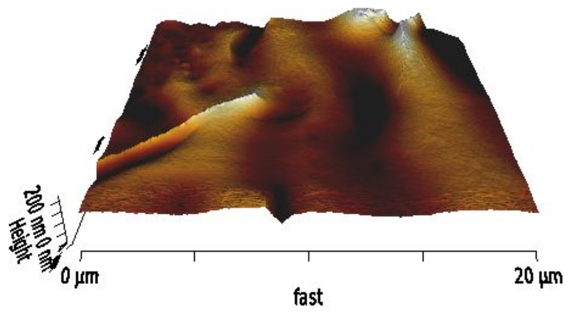

Figure S1. 20 µm × 20 µm AFM image of a PFTES film on plastic film

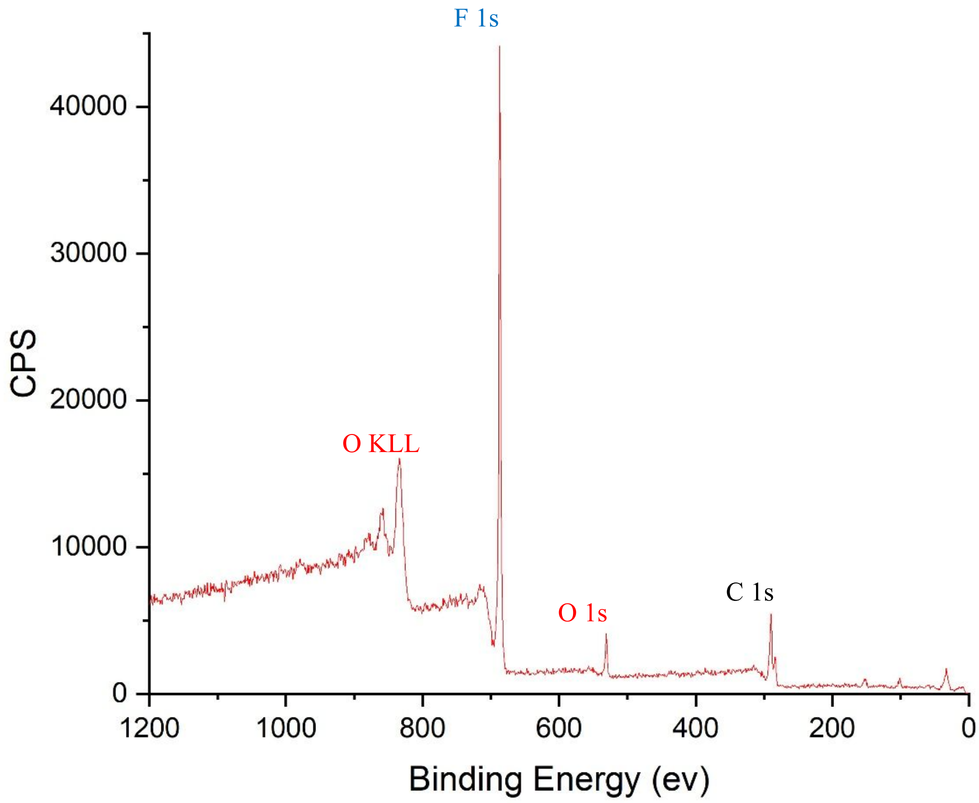

Figure S2. XPS spectrum of a PFTES film on the plastic substrate.

Table S2. Atomic percentages of elements presents in the coatings, calculated from the XPS spectra of films.

Film	Si Atomic %	C Atomic %	O Atomic %	N Atomic %
Plastic	0.00	91.52	5.70	2.78
2 TMS	5.77	75.84	18.40	0.00
4 TMS	4.09	83.67	12.25	0.00
12 TMS	7.27	80.93	11.80	0.00
16 TMS	5.83	78.03	16.13	0.00

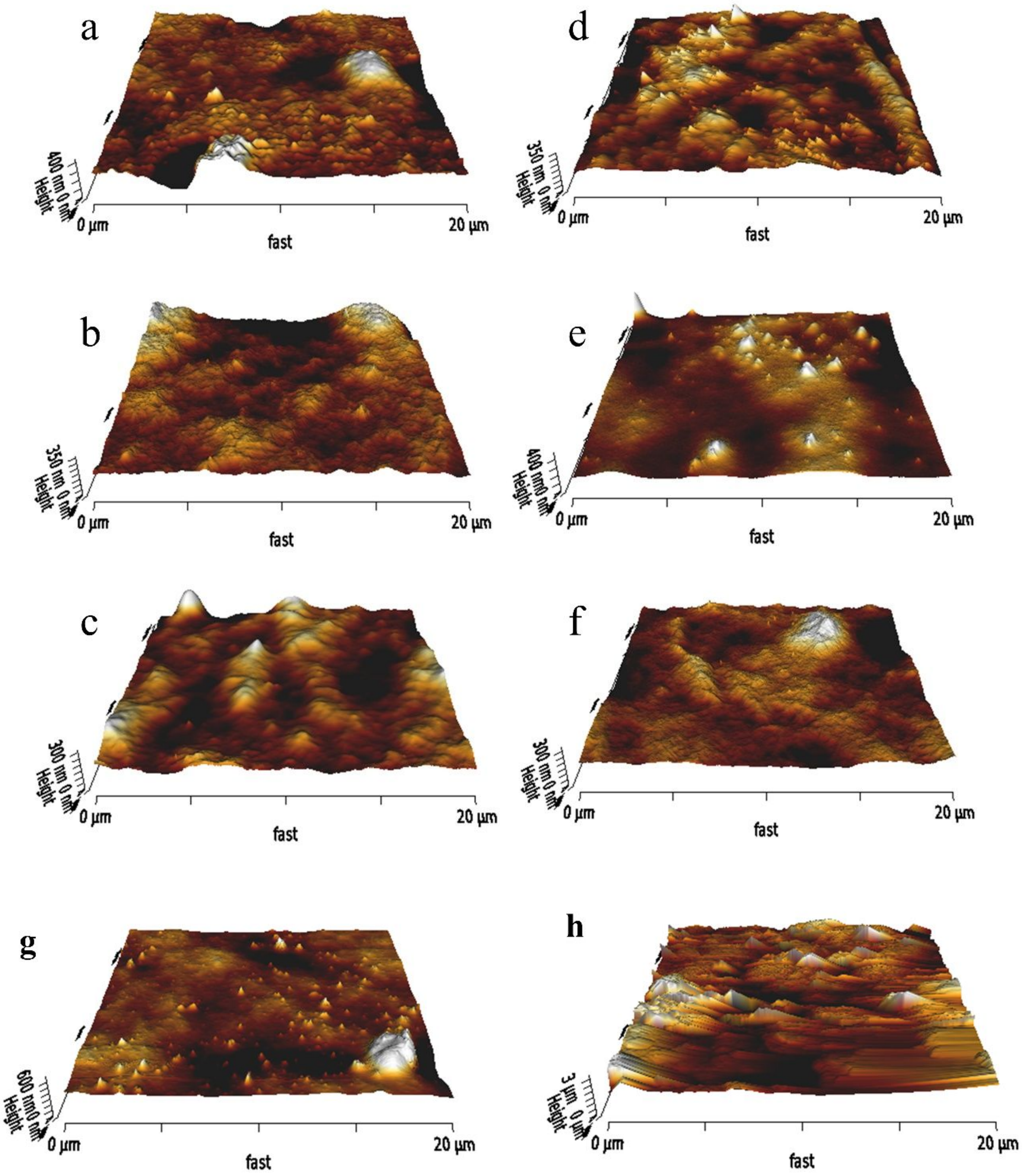

Figure S3. 20 µm × 20 µm AFM images of the a) uncoated plastic film, b) ETMS coated film c) 4 TMS coated film d) 12 TMS coated film e) 16 TMS coated film f) 18 TMS coated film g) ETMS film on plastic before annealing h) 16 TMS film on plastic before annealing.

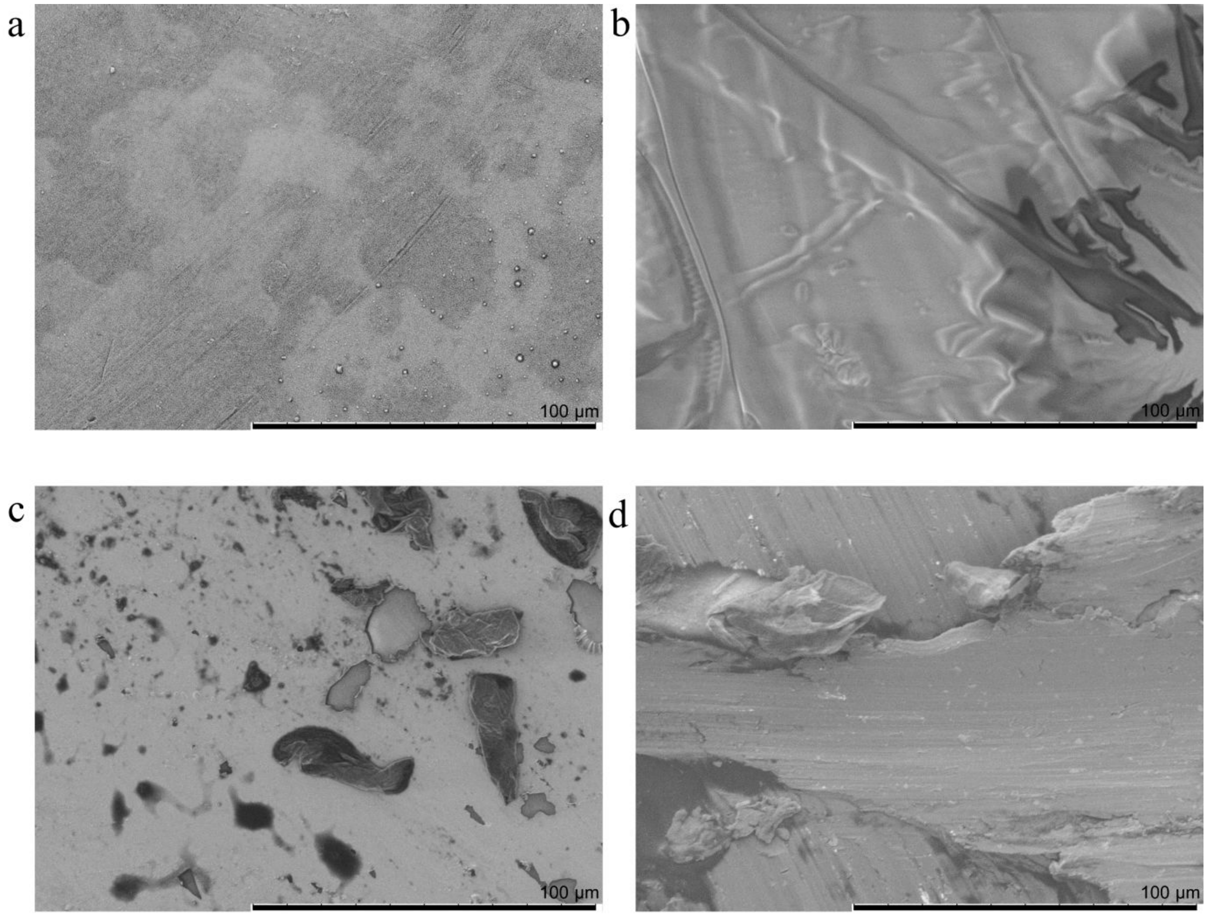

Figure S4. SEM images of glass for a) ETMS coating uncured, b) ETMS cured coated film c) 16TMS uncured coating d) 16 TMS cured coated film

Table S3. Roughness parameters obtained from 20 µm × 20 µm AFM images of the various films on the plastic substrate. (Ra- Roughness average, Rq- RMS Roughness and Rt- Maximum height of the profile.)

Surface	R_a/ nm	R_q/ nm	R_t/ nm
Plastic	48.4	66.5	681
2TMS	29.1	38.3	334.8
4TMS	27.8	36.3	320.1
12TMS	33.4	41.9	325.2
16TMS	19.9	25.4	187.7
18TMS	26.4	37.5	339.7
2TMS Uncured	39.1	56.2	728.7
16TMS Uncured	288.7	381.5	2912.6

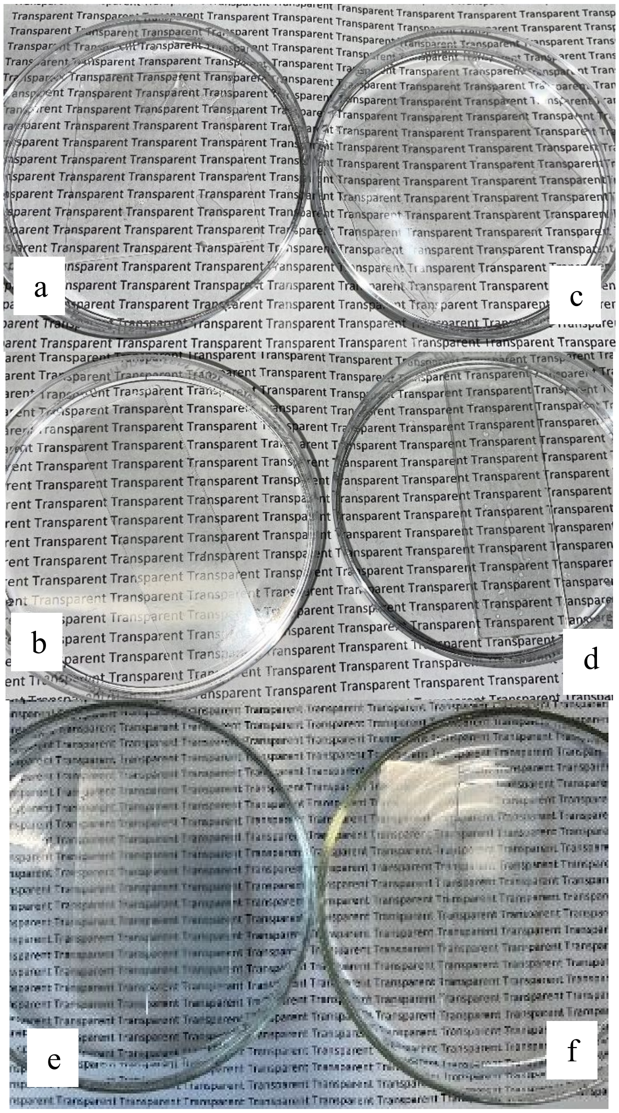

Figure S5. Photos of a range of coated films to show their transparency. (a) Non-oiled ETMS coated plastic, (b) Non-oiled ETMS coated glass, (c) Oiled 16 TMS coated plastic, (d) non-oiled 16 TMS coated plastic, (e) PFTES coated glass, (f) 10 TES coated glass.

a)

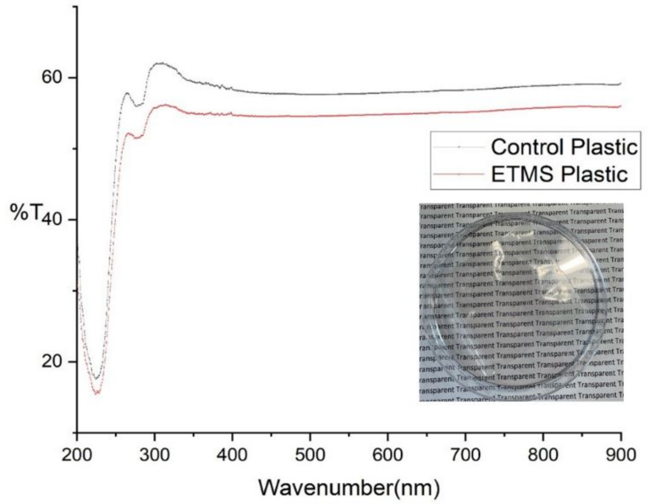

b)

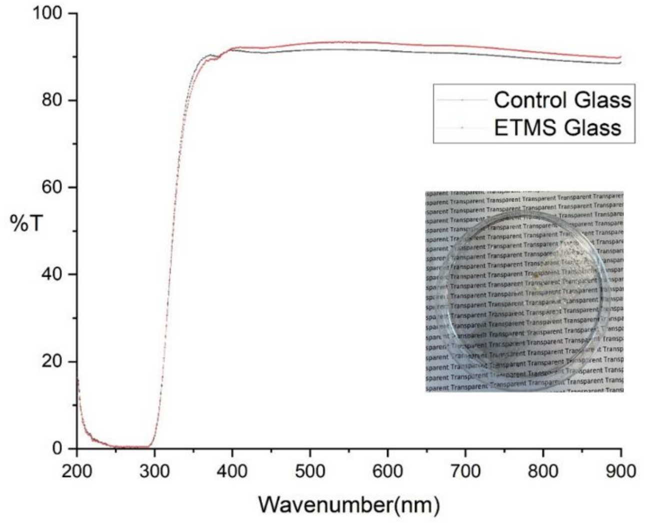

Figure S6. UV-Vis spectra of the a) plastic and b) glass samples, as received and after application of the ETMS film.

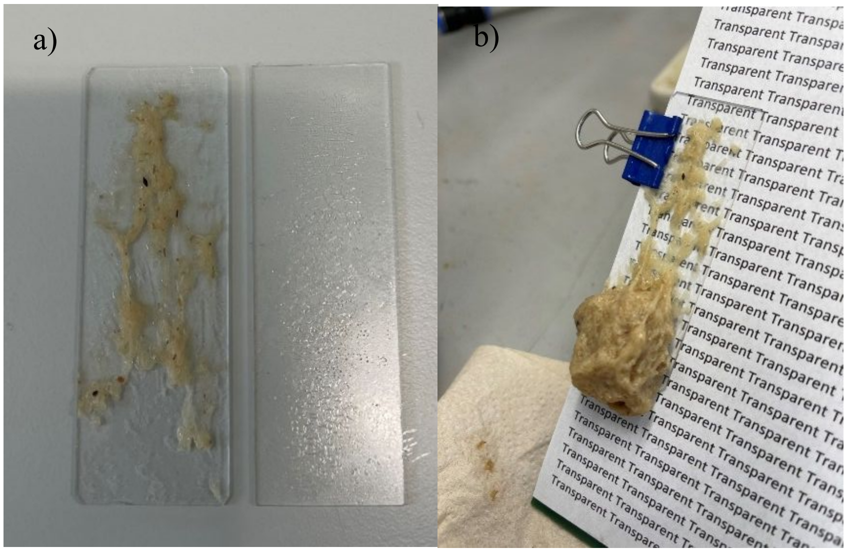

Figure S7. (a) Photos of the uncoated glass (left) and glass with a 16 TMS film (right) after application with synthetic faeces during the sliding experiments. b) Uncoated glass slide showing synthetic faeces sliding.

a)

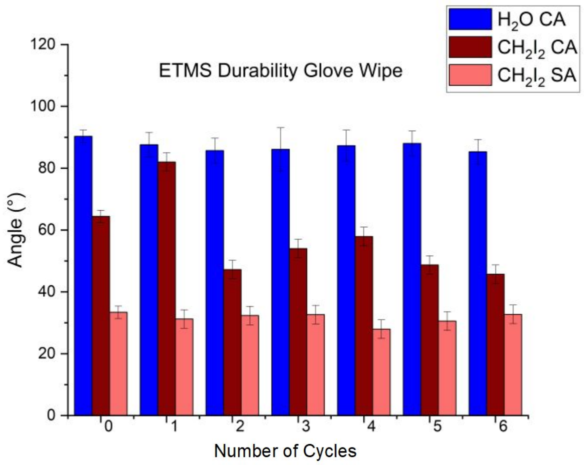

b)

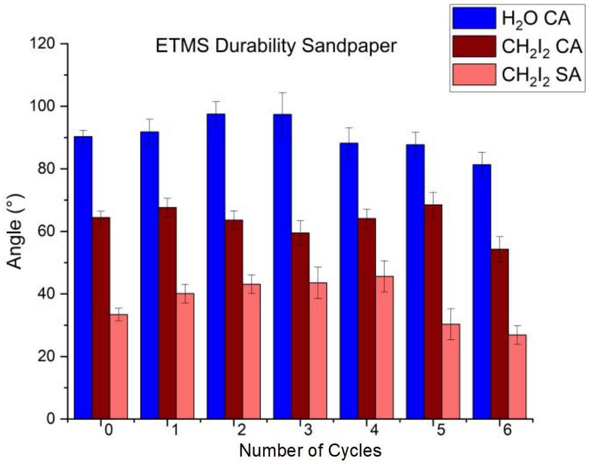

c)

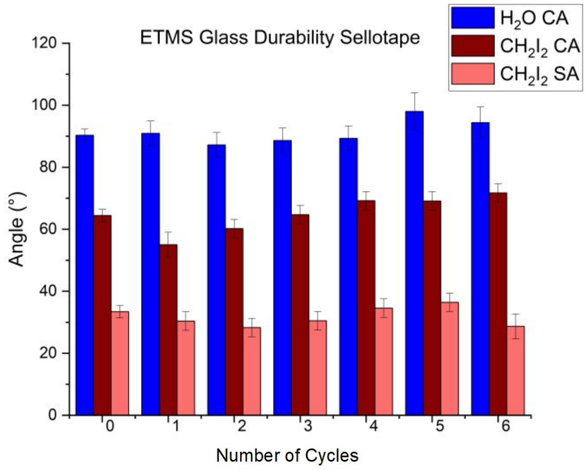

Figure S8: Graphs showing the wettability of the ETMS films after durability testing. a) repetitive wiping with a gloved finger, b) rubbing of sandpaper, and c) repetitive sticking and removal of Sellotape.

Table S4. Wettability of glass samples after application of the ETMS and 16 TMS films after different ageing times.

Film type	Time	H_2O CA/ °	H_2O SA/ °	CH_2I_2 CA/ °	CH_2I_2 SA/ °
**ETMS**	**1 Day**	106.1	-	87.6	26.7
	**1 Week**	89.3	-	79.2	23.9
	**1 Month**	75.2	-	64.5	19.9
**16 TMS**	**1 Day**	107.0	26.5	79.2	5.4
	**1 Week**	107.6	39.6	61.4	6.8
	**1 Month**	105.2	41.7	50.2	7.8

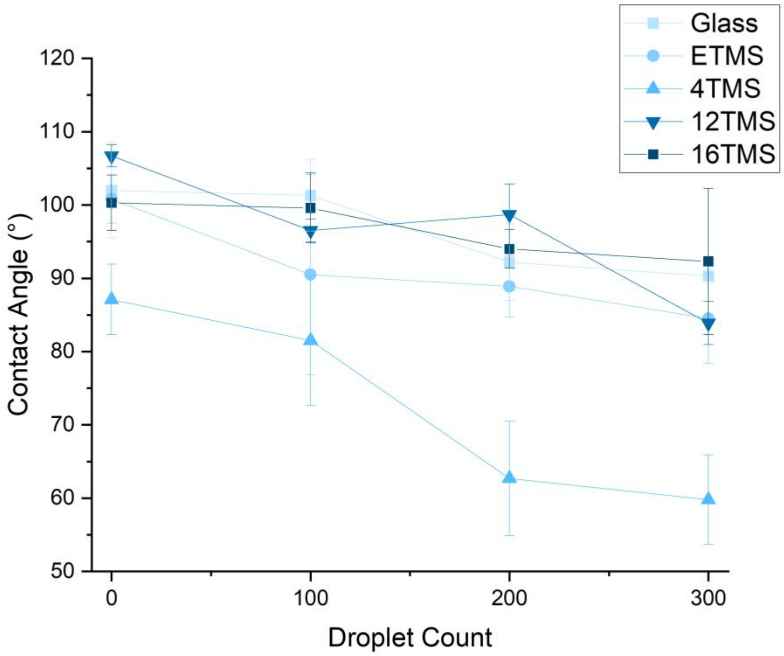

Figure S9. CAs of the various films on glass after wetting multiple droplets.
